# The Dual Burden of Malnutrition Increases the Risk of Cesarean Delivery: Evidence From India

**DOI:** 10.3389/fpubh.2018.00292

**Published:** 2018-10-17

**Authors:** Jonathan C. K. Wells, Rasmus Wibaek, Marios Poullas

**Affiliations:** ^1^Childhood Nutrition Research Centre, UCL Great Ormond Street Institute of Child Health, London, United Kingdom; ^2^Department of Nutrition, Exercise and Sports, University of Copenhagen, Copenhagen, Denmark; ^3^Clinical Epidemiology, Steno Diabetes Center Copenhagen, Gentofte, Denmark

**Keywords:** India, South Asia, dual burden of malnutrition, short stature, overweight, obesity, cesarean, obstetric dilemma

## Abstract

**Background:** Among contemporary human populations, rates of cesarean delivery vary substantially, making it difficult to know if the procedure is inadequately available, or used excessively relative to medical need. A much-cited evolutionary hypothesis attributed birth complications to an “obstetric dilemma,” resulting from antagonistic selective pressures acting on maternal pelvic dimensions and fetal brain growth during hominin evolution. However, the childbirth challenges experienced by living humans may not be representative of those in the past, and may vary in association with trends in ecological conditions. We hypothesized that variability in maternal phenotype (height and nutritional status) may contribute to the risk of cesarean delivery. In many populations, high levels of child stunting contribute to a high frequency of short adult stature, while obesity is also becoming more common. The combination of short maternal stature and maternal overweight or obesity may substantially increase the risk of cesarean delivery.

**Methods:** Using data from two large Indian health surveys from 2005–6 to 2015–2016, we tested associations of maternal somatic phenotype (short stature, overweight) with the risk of cesarean delivery, adjusting for confounding factors such as maternal age, birth order, rural/urban location, wealth and offspring sex.

**Results:** Secular trends in maternal body mass index between surveys were greater than trends in height. Maternal short stature and overweight both increased the risk of cesarean delivery, most strongly when jointly present within individual women. These associations were independent of birth order, wealth, maternal age and rural/urban location. Secular trends in maternal phenotype explained 18% of the increase in cesarean rate over 10 years.

**Conclusion:** Our results highlight how the emerging dual burden of malnutrition (persisting short adult stature which reflects persistent child stunting; increasing overweight in adults) is likely to impact childbirth in low and middle-income countries.

## Introduction

Worldwide, several hundred thousand women die annually on account of pregnancy and childbirth, while tens of millions of women suffer from serious long-term complications following parturition (1). Providing prompt access to comprehensive obstetrical emergency care is crucial to mitigate these burdens, especially in low, and middle-income countries (2). Amongst the key procedures is delivery by cesarean section (C-section) to address obstructed labor.

However, whether the rate of C-sections is well matched to medical need is subject to growing concern. Rates are rising steadily in many countries, and exceed 40% of all deliveries in several South American countries (3). On the one hand, very low rates may indicate a lack of provision of appropriate surgical care (4), while on the other, high rates suggest the influence of non-medical factors, such as high maternal socio-economic status, cultural norms, private medical care and insurance, “defensive medicine” by clinicians to mitigate the risk of malpractice, and increasing application of technology in obstetrics (3, 5).

Some studies suggest that rates >10% make no further reductions to mortality, and that mortality increases again above rates of 15% (6). Among women experiencing low-risk pregnancies, C-sections are associated with greater risk of birth complications, maternal mortality and longer-term maternal morbidity (7). Offspring delivered by C-section miss the hormonal exposures and mother-offspring microbial exchange associated with vaginal delivery (8), while the process is also associated with metabolic and immune diseases in later life (9). The World Health Organization (WHO) has proposed that C-sections should not exceed 10–15% of total deliveries, and should only be used when health or life of the mother or newborn is at risk (2). However, other studies suggest that rates up to 19% may still reduce maternal or neonatal mortality (10).

The “optimal” rate of C-sections therefore remains controversial, and aside from contrasting societal trends it is possible that biological risk factors also differ between populations. Here, we focus on maternal somatic phenotype, which has been linked previously with variability in delivery experience (11). Maternal somatic phenotype varies substantially within and between populations, and can also change over time through secular trends in nutrition and growth (12).

### An evolutionary perspective

Our understanding of the relationship between maternal phenotype and the challenges of childbirth has been powerfully shaped by an evolutionary perspective, which focused on the problem at the level of the entire human species. Hominin evolution demonstrates the emergence of two key characteristics—bipedal locomotion and increased brain size—that have mutual implications for each other, because under natural conditions the fetal head must pass through the maternal pelvis at birth (13). Birth in humans is a complex process, and humans are unusual in the near-universal tendency for women to seek assistance during delivery (14). In the 1960s, Washburn described these challenges as the “obstetrical dilemma” (OD), and suggested that antagonistic selective pressures constrained the size of the maternal pelvis in order to maintain efficient locomotion, while also favoring larger fetal head size in order to maximize prenatal encephalization (15). Washburn proposed that selection had favored a partial resolution of this antagonism by favoring a relatively shorter gestation of humans relative to other primates, but childbirth complications and persisting maternal mortality were considered additional adverse consequences.

Recently, the OD proposed by Washburn has been extensively critiqued. Maternal pelvic dimensions vary substantially within and across populations, with no obvious impact on maternal locomotory biomechanics (16). Moreover, it is increasingly recognized that the nature of the OD may change over time, through both natural selection and phenotypic plasticity (17, 18). The tight fit between the maternal pelvis and fetal size within individual dyads therefore requires alternative explanation.

The OD may be reconsidered as a “coordination problem” (18) regarding the dimensions of the maternal pelvis and fetus. From a genetic perspective, delivery is characterized by the interaction of contrasting fitness functions. The discrepancy between pelvic and fetal dimensions demonstrates a normal distribution, however individual female fitness demonstrates a “cliff-edge” form, because delivery becomes impossible once fetal size exceeds a certain threshold (19). On this basis, it is inevitable that the phenotypic distribution that maximizes population mean fitness is associated with a proportion of individuals exceeding the “cliff-edge,” and thus being too large for natural delivery (19). Large differences between maternal and paternal size may exacerbate this scenario (20).

Aside from genetic factors, however, phenotypic plasticity in both mother and fetus must also be considered (11, 17, 18). The dimensions of the maternal pelvis reflect environmental conditions and nutritional experience during the mother's own development, and secular trends in maternal height extend to pelvic dimensions (21). This inherently acts against a strong genetic influence on fetal growth, and instead favors fetal growth being responsive to maternal somatic and metabolic phenotype (18, 22). Crucially, the environmental factors that impacted growth of the maternal pelvis may be very different from those impacting fetal growth, since they broadly occur one generation apart in time (18). The risk of feto-pelvic disproportion may be exacerbated, should ecological conditions change substantially across the maternal life-course.

Independently, both maternal short stature and maternal overweight/obesity (categorized by body mass index, BMI) have been widely associated with an increased risk of C-section, with obstructed labor a key underlying factor (11). The underlying reasons are well established: on the one hand, short stature indicates a smaller pelvis, while on the other, maternal obesity is associated with greater fetal weight gain (11). Both traits are therefore predicted to increase the risk of feto-maternal disproportion.

However, research to date has tended to target only one of these traits at a time. It might be assumed that short stature and overweight afflict different populations, with contrasting environmental conditions and little overlap in geographic distribution. However, an increasing proportion of women in low- and middle-income countries are characterized by both nutritional states, having become stunted in early life, and then developed overweight/obesity subsequently. This individual manifestation of the “dual burden” of malnutrition (23, 24) can be attributed to the global obesity epidemic emerging faster than child under-nutrition (childhood stunting, leading to short adult stature) is being resolved (11).

### Hypothesis

The dual burden of malnutrition is predicted to exacerbate the risk of feto-pelvic disproportion, but this issue has received little attention. The study aims were threefold: (i) to document secular trends in the rate of C-section and in maternal and offspring variables over a 10 year period in India, (ii) to test for associations of maternal somatic phenotype (short stature, overweight and obesity) with the risk of C-section, and (iii) to establish how the emerging dual burden of malnutrition (persistent short stature, rising levels of overweight and obesity) might be driving any secular increase in C-section rate. We hypothesized that short women of normal BMI, and overweight or obese women, would each have an increased risk of C-section, and that this risk would increase further if women were both short and overweight. We further hypothesized that rising levels of overweight would contribute to secular increases in C-section rate over time.

## Materials and methods

To test these hypotheses, we conducted cross-sectional analysis of data from two DHS surveys from India (2005–2006 and 2015–2016). The data was downloaded from the MEASURE DHS website (www.dhsprogram.com). Each survey records data related to children aged < 5 years, allowing us to incorporate births over the last 5-year period in the analysis.

These data derive from nationally representative cross-sectional household surveys, incorporating detailed birth histories for women of reproductive age. Analysis of these surveys enabled us to document secular trends both in C-section rates, maternal somatic phenotype and offspring birth size, and potential confounders such as maternal age, birth order, wealth status, rural-urban age, birth order, and wealth with C-section rate. These analyses were all restricted to live singleton births. In each survey separately, we explored associations of maternal stature and BMI with C-section rate, adjusting for confounders. We then analyzed both surveys combined, in order to generate a more accurate assessment of the secular increase in C-section rate, and the extent to which secular trends in maternal and offspring size contributed to it.

All procedures and questionnaires for standard DHS surveys have been reviewed and approved by ICF Institutional Review Board (IRB). Additionally, country-specific DHS survey protocols are reviewed by the ICF IRB and typically by an IRB in the host country. ICF IRB ensures that the survey complies with the U.S. Department of Health and Human Services regulations for the protection of human subjects (45 CFR 46), while the host country IRB ensures that the survey complies with laws and norms of the nation. Before each interview is conducted, an informed consent statement is read to the respondent, who may accept or decline to participate. A parent or guardian must provide consent prior to participation by a child or adolescent.

The surveys provide data on maternal anthropometry (weight and height, measured by field staff using standardized protocols and equipment: Seca 874 digital scales and a Shorr height board). Weight is measured in light clothing after removal of shoes/sandals and any heavy clothing, while height is measured with the measuring board on a flat surface, feet and shoulders in standardized position, and head in the Frankfort plane (25). These data allow the calculation of body mass index (BMI) in kg/m^2^. As there is no specific cut-off for short stature in adults, and because adult stature varies substantially across populations, we used a threshold of 148 cm, roughly equivalent to the shortest quarter (24.9%) of the population. For BMI, we used cut offs for overweight and obesity of 23 and 27 kg/m2 respectively, similar to cut-offs proposed for Asian populations (23 and 27.5 kg/m^2^) but with a slightly lower cut-off for obesity to increase the sample size (26). Mothers were also asked if they had diabetes, and responded yes, no or don't know.

Offspring were not routinely weighed at birth, however a 5-point abstract score was provided, comprising “very small,” “smaller than average,” “average,” “larger than average” and “very large.” We created a binary variable, differentiating large offspring (“larger than average” and “very large”) from the other categories.

We restricted analysis to singletons, excluding 3,245 offspring of multiple births. Birth order ranged from 1 to 14. We included women who had given birth to 3 or fewer singleton offspring within the 5-year period (thus excluding 0.1% of mothers in each survey who had delivered 4+ children). As the number of women with individual birth order categories was small (*n* < 2,500) for each birth order category >6, we grouped together all women with birth order ≥6 into a single category (*n* = 9,300, 4.0% of the sample).

The survey recorded whether a birth was by C-section or not, and the location of the birth. The specific question was: “Was (NAME) delivered by cesarean, that is, did they cut your belly open to take the baby out?” We screened the data for locations where a C-section was implausible (e.g., a home birth) but no such cases were detected.

Socioeconomic status was assessed as a relative wealth index based on household assets, calculated using principal components analysis. Each household was categorized by wealth quintile, categorized as poorest, poorer, middle, richer, richest. The survey also recorded whether the location was rural vs. urban.

### Survey reliability

DHS surveys are generally regarded as reliable data sources for assessing secular trends in anthropometric and birth outcomes, and have been used in similar analyses of obesity and cesarean trends previously (27–30). Regarding the 2015–2016 survey in India, there were multiple levels of monitoring and supervision of the fieldwork. The field supervisor on each interviewing team observed interviews in a subsample of households and conducted back-checks with respondents as a further check on fieldwork quality. A standard set of 42 field-check tables were produced frequently throughout the fieldwork, covering such topics as response rates, age heaping and age displacement, completeness of reporting, and patterns of anthropometric measurements. Regarding sampling, 628,900 households were selected for the survey, of which 616,346 were occupied, of which 601,509 were successfully interviewed, giving a response rate of 98 percent. Within this sample, 5.6% of women had missing weight or height data (25).

Assessing reliability of DHS questionnaire data on cesarean delivery, two key issues were identified (31). First, it is important that the questionnaire clearly enquires about cesarean delivery, rather than delivery complications in general. Second, screening is recommended for implausible locations of cesarean delivery, i.e., locations without appropriate medical facilities. Both of these criteria were met in the two surveys analyzed, as described above.

### Statistical analysis

We excluded maternal stature values >5 standard deviations from the population mean (< 120 or >180 cm; 2005: *n* = 13; 2015: *n* = 477). On a similar basis, mothers with BMI >45 kg/m^2^ (2005, *n* = 6; 2015, *n* = 101) were also excluded. Low BMI values were not excluded as the lowest values did not appear to represent a separate distribution from the overall sample.

We first quantified median maternal height and BMI by age category in the 2005–2006 survey. We then estimated the increment in maternal height and BMI in each age group in the 2015–2016 survey, using multiple regression analysis with a dummy variable for the second survey, while holding constant for wealth index category and rural-urban location, in order to test for secular trends in these nutritional outcomes. We also described C-section rates by maternal age, birth order and wealth index in each survey, testing for differences using chi-squared tests. We also showed graphically how secular increases in C-section rate between surveys were distributed across these categories of maternal or offspring phenotype.

We further explored potential interactions between birth order and wealth index, or between survey year and maternal age, wealth category, birth order or offspring birth size, in relation to C-section status. This was undertaken by fitting relevant interaction terms to logistic regression models, and, for the wealth-birth order association by assessing trends for one predictor stratified by categories of the other predictor.

Finally, we constructed multivariable logistic regression models to examine the associations of short stature, overweight/obesity or their combined manifestation within individual women with the odds C-section, holding constant for confounding factors. As potential confounders, we included maternal age categories, birth order category (6 groups), birth sequence within the 5 year survey period, rural/urban status, offspring sex, offspring birth size (large vs. not large) and wealth group (5 groups). These potential confounders were selected for the following reasons. Dimensions of the maternal pelvis continue to increase with maternal age after the linear growth has ceased, and they also vary in association with parity (32). Birth order associations with birth weight are also reported (33), while the risk of C-section may change within individual women across successive pregnancies. Urban populations may have greater access to medical facilities compared to rural populations. Wealth is an established predictor of C-section (3, 5).

The first logistic regression model was constructed for the 2015–2016 survey. Having quantified the associations of maternal phenotype, we then added maternal diabetes (yes/no) to test whether it was associated with risk of C-section, independent of maternal obesity. We ran the same model separately for the smaller 2005–2006 survey, to confirm the pattern of associations.

A second set of logistic regressions was then constructed, incorporating both surveys. The first of these two-survey models did not include maternal phenotype, and quantified the increased risk of C-section in the second survey compared to the first, adjusting for confounders. Maternal phenotype was then added to this model, to establish how much the survey coefficient declined, and hence how much of the secular increase in C-section rate was due to secular changes in maternal somatic phenotype. We did not include offspring size in this model as it mediates the association between secular trends in maternal phenotype and C-section rate.

Given that wealth is associated with both maternal stature and BMI, we further considered whether associations of maternal phenotype and the rate of C-section were evident within each of the five wealth categories. We first described crude C-section rates by maternal phenotype for each wealth group, and then for each individual wealth category, we applied the logistic regression model described above to quantify the association of maternal phenotype with C-section risk, independent of other confounders.

All data analyses were performed in SPSS (Version 24, IBM Corporation).

## Results

The 2005–2006 survey included 31,949 mothers, of whom 31,695 (99.2%) were retained following exclusions and who contributed 42,869 births eligible for analysis. The 2015–2016 survey included 177,600 mothers, of whom 175,790 (99.0%) were retained and contributed 232,411 births eligible for analysis. Across the two surveys combined, the majority of the women (75.4%) contributed 1 birth to the analysis, 22.2% contributed 2 births, and 2.4% contributed 3 births. Basic characteristics of the two samples are given in Table 1, which also reports differences between the two surveys in the frequencies of various variables, assessed by chi-squared test. Compared to the 2005–2006 survey, the 2015–2016 survey sampled a higher proportion of households in rural locations and of poorer wealth status, a greater proportion of mothers were in higher age groups, and a greater proportion of offspring were first- or second-born (all *p* < 0.0001 by chi-squared test).

**Table 1:** Comparison of sample characteristics between the two DHS surveys.

	**2005–2006 survey (*n* = 42,869)**		**2015–2016 survey (*n* = 232,411)**		
	**Number**	**%**	**Number**	**%**	**P for Chi-squared^a^**
**MATERNAL AGE (Y)**					
15–19	2,156	5.0	5,859	2.5	<0.0001
20–24	13,829	32.3	70,178	30.2	
25–29	14,931	34.8	89,459	38.5	
30–34	7,768	18.1	43,832	18.9	
35–39	3,118	7.3	16,938	7.3	
40–44	858	2.0	4,775	2.0	
45–49	209	0.5	1,370	0.6	
**LOCATION**					
Rural	27,121	63.3	177,339	76.3	<0.0001
Urban	15,478	36.7	55,072	23.7	
**BIRTH ORDER**					
1	13,631	31.8	86,165	37.1	<0.0001
2	12,165	28.4	72,077	31.0	
3	6,981	16.3	37,251	16.0	
4	4,176	9.7	18,490	8.0	
5	2,522	5.9	9,128	3.9	
6+	3,394	7.9	9,300	4.0	
**WEALTH INDEX**					
Poorest	7,619	17.8	60,816	26.2	<0.0001
Poorer	7,919	18.5	54,934	23.6	
Middle	8,943	20.9	46,443	20.0	
Richer	9,489	22.1	38,816	16.7	
Richest	8,899	20.8	31,402	13.5	
**OFFSPRING SEX**					
Male	22,358	52.2	120,931	52.0	0.6
Female	20,511	47.8	111,480	48.0	

a *Chi-squared test to compare the sample distribution between the two surveys*.

### Secular trends in maternal and offspring size across the surveys

**Figure 1:**
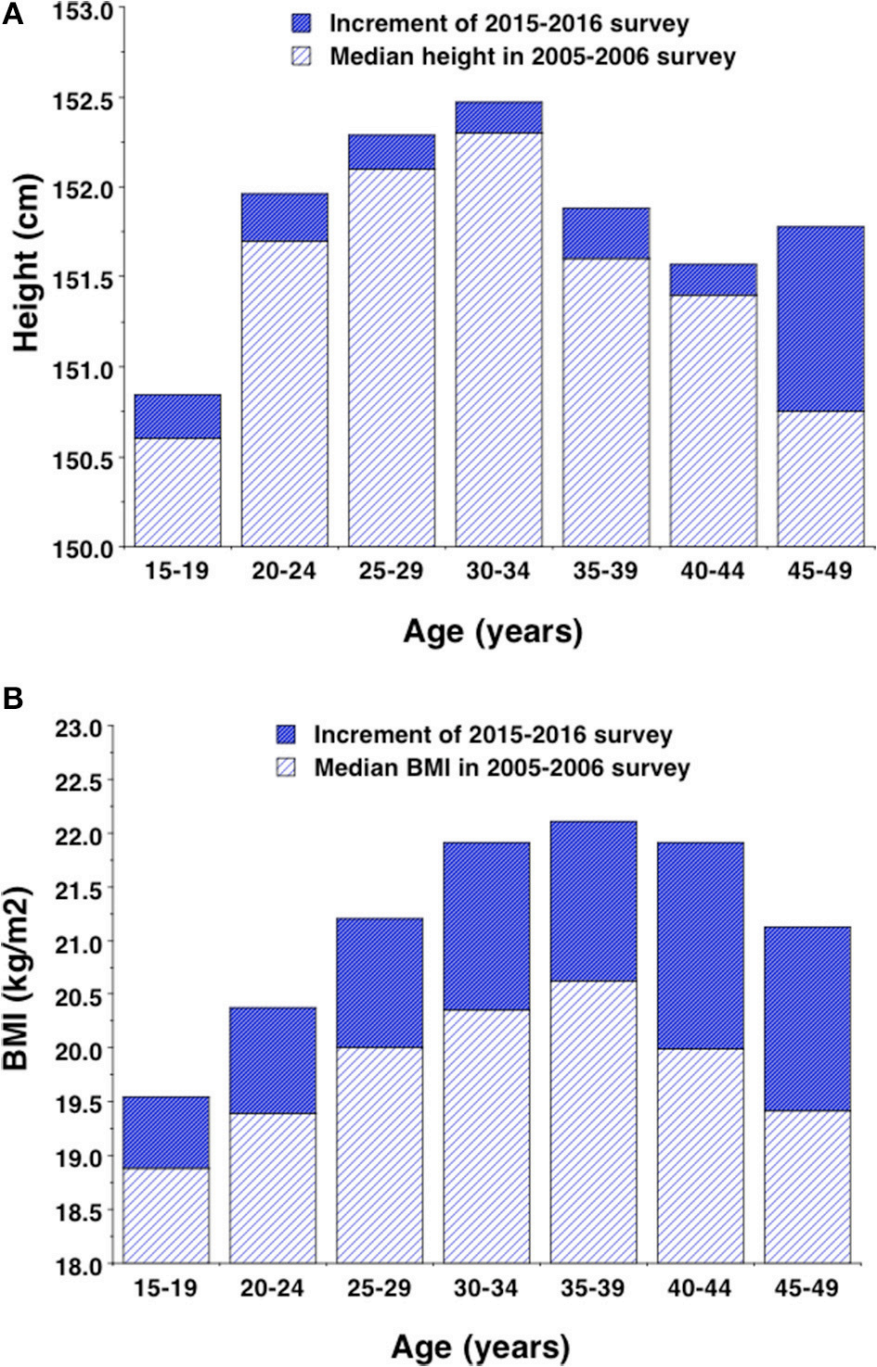
Median values for **(A)** height and **(B)** body mass index by 5-year age group in the 2005–2006 survey, with the increment demonstrated by the 2015–2016 survey. Increments are adjusted for wealth and rural-urban location so as to match the profile of the baseline survey.

Associations of maternal height and BMI with age category are given in Figure 1. These trends were broadly consistent across the two surveys, but with consistently higher values in the second survey. Median maternal height in the 2005–2006 survey increased in association with age up until around 30–34 years, and then declined with age from the mid-30s. Adjusting for wealth category, rural/urban location and age, height in the more recent survey was 0.25 cm higher (95%CI 0.18, 0.32), with this increment relatively consistent across the range of maternal age but substantially greater in the oldest age category (Figure 1A). Median maternal BMI in the 2005–2006 survey increased systematically with age up until around 35–39 years, and then fell with older age. Adjusting for wealth category and rural/urban location, maternal BMI was greater in the second survey (Δ = 1.28 kg/m^2^, 95%CI 1.24, 1.32), with this increment increasing in association with maternal age (Figure 1B).

Table 2 reports proportions of women in different categories of nutritional status in the two surveys, comparing between the surveys by chi-squared test. The proportion of women with normal BMI and height was 6.1% lower in the more recent survey, due almost entirely to increases in the proportion of those overweight or obese, whereas the total proportion categorized as short was actually greater in the second survey (25.5 vs. 24.2%). The two forms of malnutrition co-occurred in 3.8% of women in the 2005–2006 survey, but in 5.8% of women in the 2015–2016 survey. Table 2 also provides the proportions of offspring in the five categories of birth in each survey. The 2015–2016 survey showed a lower percentage of “very small,” “smaller than average” and “larger than average” offspring than the 2005–2006 survey, and a greater percentage of “average” size and “very large offspring.”

**Table 2:** Comparison of maternal somatic phenotype categories, and infant birth size categories, between surveys.

	**2005–2006 survey**		**2015–2016 survey**		**P for Chi-squared^a^**
	**Number**	**%**	**Number**	**%**	
**MATERNAL PHENOTYPE**					
Normal height and BMI	26,322	61.3	128,426	55.2	<0.0001
Normal height, overweight	4,457	10.4	33,010	14.2	
Normal height, obese	1,762	4.1	13,269	5.7	
Short, normal BMI	8,782	20.4	45,548	19.6	
Short, overweight	1,223	2.9	9,073	3.9	
Short, obese	387	0.9	3,338	1.9	
**OFFSPRING BIRTH SIZE**					
Very small (%)	2,381	5.5	6,208	2.7	<0.0001
Smaller than average (%)	5,995	14.0	20,332	8.7	
Average (%)	24,207	56.4	161,448	69.4	
Larger than average (%)	8,083	18.8	28,044	12.1	
Very large (%)	1,583	3.7	11,513	5.0	
No data (%)	660	1.6	5,087	2.2	

a *Chi-squared test to compare the sample distribution between the two surveys*.

### Secular trends in c-section rates and associations with confounders

**Figure 2:**
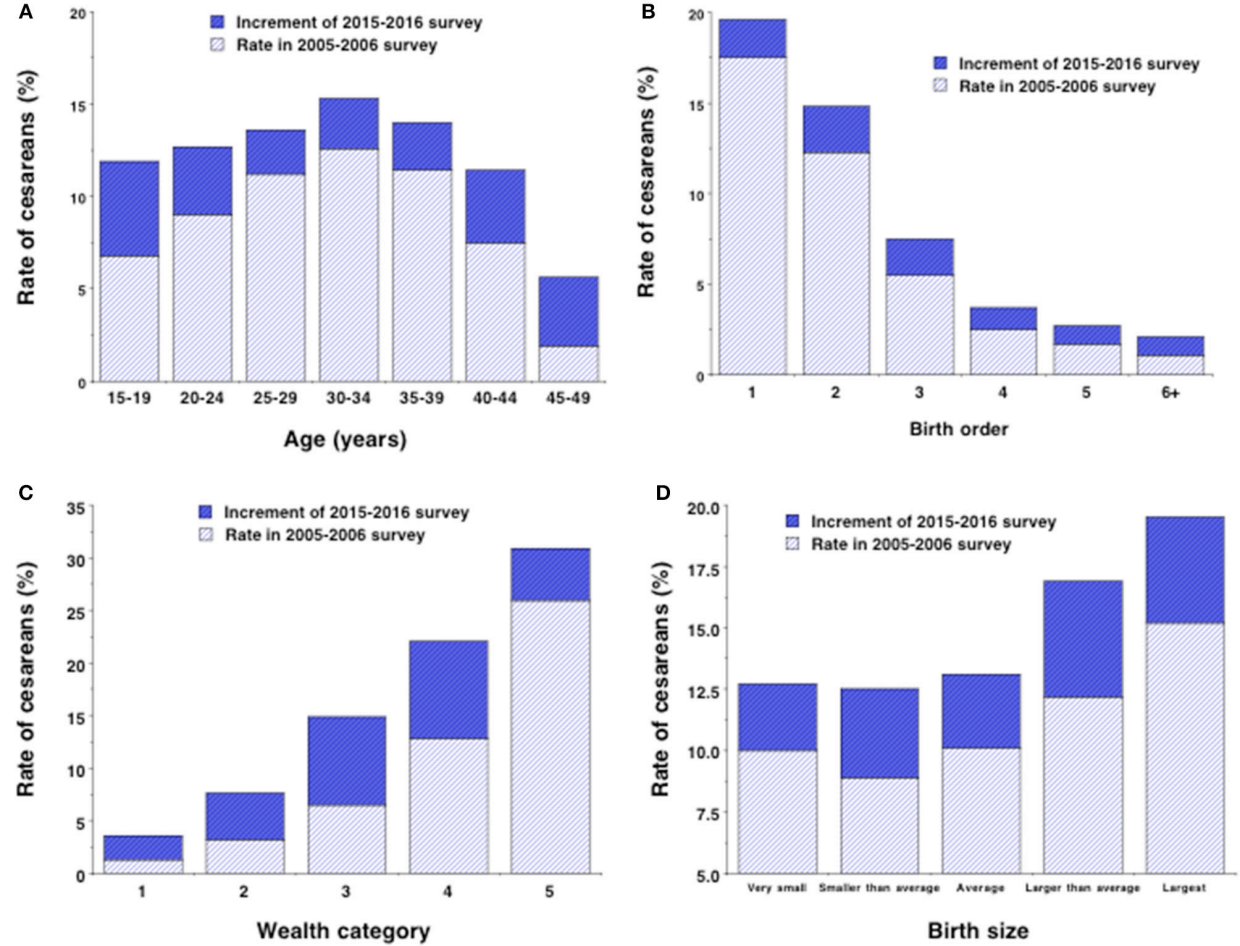
Rates of C-section stratified by **(A)** 5-year age group, **(B)** birth order, **(C)** wealth category group (5 = wealthiest) and **(D)** infant birth size in the 2005–2006 survey, with the increment in the rate demonstrated by the 2015–2016 survey.

Overall, crude C-section rate increased from 10.4 to 13.6% across the two surveys, with this secular increase greater in younger and older mothers compared to those in the middle of the age range. In each survey, C-section rate increased in association with maternal age from 15–19 years to 30–34 years and then fell beyond this to so that the lowest rates occurred among those aged 45–49 years (Figure 2A; *p* < 0.0001 for overall age-survey interaction).

In each survey, C-section rate fell strongly in association with birth order category, with values for the 2015–2016 survey declining from 19.6% among firstborn offspring to 2.1% among birth order 6+ (Figure 2B). The increase in C-section rate across surveys occurred disproportionately in those of birth order 1 to 3 (>2%) compared to those of higher birth order (< 1.25%; *p* < 0.0001 for interaction).

C-section rate rose strongly in association with wealth index, with values for the 2015–2016 survey rising from 3.6% among the bottom category to 30.9% among the top category (Figure 2C). The secular increase in C-section rate across surveys was greater in wealth categories 3 and 4 (>8%) than in categories 1, 2, or 5 (< 5%; *p* < 0.0001 for interaction).

C-section rates increased with offspring birth size, but were also systematically higher in the second survey, more so in the two largest birth size categories (Δ ≥ 4.3%) than in the three smaller size categories (Δ ≤ 3.6%; *p* = 0.041 for interaction) (Figure 2D).

**Figure 3:**
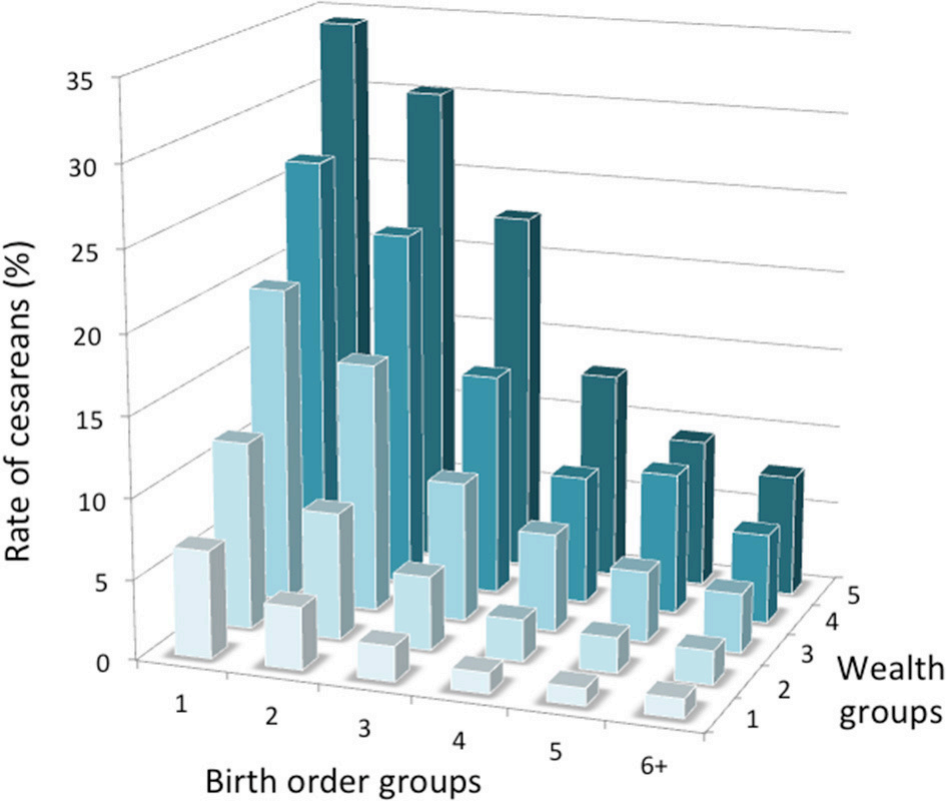
Interactive associations of birth order and wealth category with the rate of C-section in the 2015–1016 survey (5 = wealthiest). Numbers per group are given in Table S3.

The associations of birth order and wealth category with C-section rate were independent and interactive (*p* < 0.0001), as shown in Figure 3 for the 2015–2016 survey. Within each wealth category, C-section rate fell with increasing birth order (*p* < 0.0001 in all cases), while within each birth order group, C-section rate rose with increasing wealth (*p* < 0.0001 in all cases). Thus, first-time mothers in the richest wealth group had a C-section rate of 34.3%, whereas mothers in the poorest group with birth order 6+ had a rate of only 1.2%. Wealth showed a weak association with C-section rate in high-parity women but a very strong association in first-time mothers (*p* < 0.0001 for interaction). Likewise, birth order showed a weak association with C-section rate in poor women but a very strong association in rich mothers (*p* < 0.0001 for interaction).

In both surveys, C-section rate was greater among urban than rural populations (2005–2006: 17.6 vs. 6.2%; 2015–2016: 23.2 vs. 10.5%; both *p* < 0.0001 by chi-squared test). The association of rural/urban location with C-section rate was mediated by wealth status. For example, in the poorest group of rural women in the 2015–2016 survey, only 3.5% delivered by C-section, whereas amongst the wealthiest group of urban women, 33.4% delivered by C-section.

In both surveys, C-section rate was greater among mothers producing sons than daughters (2005–2006: 10.8 vs. 10.0%; 2015–2016 survey, 14.0 vs. 13.1%; both *p* < 0.0001). Mothers of sons were also more likely to be in the rich wealth group and less likely to be in the poor wealth group (*p* < 0.0001).

### Crude associations of maternal phenotype with confounders (2015–2016 survey)

Rates of maternal short stature fell from 30.6 to 5.5% across the five wealth categories, whereas maternal overweight increased from 9.3 to 30.6% and maternal obesity from 1.5 to 19.3%. The proportion of women both short and overweight/obese was < 8% in any individual wealth category, and tended to increase in association with wealth, more so for obesity than overweight (Table S1). The distribution of confounding factors also varied across the wealth categories, with the wealthiest women being the least likely to be aged 15–19 years, most likely to be living in an urban location, most likely to be a first time mother, and most likely to deliver a son (Table S1).

Rural populations showed higher rates of short stature (26.2 vs. 20.7%, *p* < 0.0001) than urban populations and lower rates of overweight (15.7 vs. 25.5%, *p* < 0.0001) and obesity (5.0 vs. 13.9% *p* < 0.0001). Among the poorest rural women (*n* = 58,175), 34.8% had short stature while 10.1% were either overweight or obese, and 3.6% were both short and overweight/obese. Among the wealthiest urban women (*n* = 19,436), 12.3% had short stature while 51.9% were either overweight or obese, and 6.8% were both short and overweight/obese.

The proportion of overweight and obese women having an offspring categorized “larger than average” or “very large” at birth (18.7 and 21.1% respectively) was greater than that for women with normal BMI (16.7%). A higher proportion of short women than taller women had a “very small” or “smaller than average” baby (13.8% vs. 11.0%, *p* < 0.0001), but the proportion decreased from 14.3% among short women with normal BMI to 12.3% for short overweight women and 11.0% for short obese women. Similarly, short women were less likely than taller women to have a “larger than average” or “very large” baby (16.0 vs. 17.8%, *p* < 0.0001), but the proportion increased from 15.7% among short women with normal BMI to 16.7% for short overweight women and 19.5% for short obese women.

### Adjusted associations of maternal and offspring phenotype with c-section rate

Table 3 provides the multiple logistic regression model, testing associations of maternal phenotype with the odds of delivering by C-section, taking into account the confounders identified above. In the adjusted model, the risk of C-section broadly increased in association with maternal age, being greatest for the age group 40–44 years compared to the reference group 15–19 years. The risk of C-section increased progressively through the wealth groups, being 4.8 times greater in the richest compared to the poorest groups, and independent of that was 18% greater in urban compared to rural populations. The risk of C-section increased progressively with lower birth order, being 14 times greater in first time mothers compared to those whose offspring was birth order 6+. The risk of C-section was also greater for the most recent birth, compared to earlier births within the survey period. Mothers of male offspring were 3% more like to deliver by C-section than mothers of female offspring. Very similar findings were apparent in the smaller 2005–2006 survey (Table S2).

**Table 3:** Multivariable logistic regression model for the odds of cesarean delivery in the 2015–2016 survey in association with maternal somatic phenotype, (a) without and (b) with adjustment for covariates.

		**(a) Unadjusted for covariates**			**(b) Adjusted for covariates**		
		**Nagelkerke *r*^2^ = 0.071**			**Nagelkerke *r*^2^ = 0.218**		
**Predictor**	***N***	**OR**	**95% CI**	***p***	**OR**	**95% CI**	***p***
**MATERNAL NUTRITIONAL STATUS**							
Normal height normal BMI (ref)	125,485	1.0	–	–	1.0	–	–
Normal height, overweight	32,441	2.41	2.33, 2.49	<0.0001	1.65	1.60, 1.71	<0.0001
Normal height, obese	13,135	5.15	4.95, 5.36	<0.0001	3.01	2.88, 3.15	<0.0001
Short, normal BMI	44,146	0.95	0.91, 0.98	0.004	1.38	1.33, 1.44	<0.0001
Short, overweight	8,833	2.33	2.21, 2.46	<0.0001	2.27	2.14, 2.41	<0.0001
Short, obese	3,289	4.83	4.49, 5.20	<0.0001	3.49	3.22, 3.79	<0.0001
**OFFSPRING BIRTH SIZE**							
Average size or smaller (ref)	187,808				1.0	–	–
Larger than average or very large	39,521				1.33	1.29, 1.38	<0.0001
**MATERNAL AGE**							
15–19 years (ref)	5,726				1.0	–	–
20–24 years	68,961				1.15	1.05, 1.25	0.0020
25–29 years	87,680				1.46	1.34, 1.59	<0.0001
30–34 years	42,784				2.09	1.91, 2.29	<0.0001
35–39 years	16,345				2.67	2.41, 2.94	<0.0001
40–44 years	4,551				3.21	2.80, 3.68	<0.0001
45–49 years	1,282				2.55	1.93, 3.37	<0.0001
**BIRTHS WITHIN SURVEY**							
Most recent birth within 5 years (ref)	172,313				1.0	–	–
2nd most recent birth within 5 years	49,711				0.49	0.47, 0.51	<0.0001
3rd most recent birth within 5 years	5,305				0.21	0.18, 0.25	<0.0001
**WEALTH INDEX**							
Level 1 (ref)	58,585				1.0	–	–
Level 2	53,302				1.80	1.70, 1.90	<0.0001
Level 3	45,666				3.11	2.95, 3.27	<0.0001
Level 4	38,497				4.02	3.82, 4.24	<0.0001
Level 5 (wealthiest)	31,279				4.83	4.57, 5.11	<0.0001
**RESIDENCE**							
Rural (ref)	172,822				1.0	–	–
Urban	54,507				1.18	1.15, 1.22	<0.0001
**BIRTH ORDER**							
First-born	85,028				14.39	12.3, 16.8	<0.0001
Second-born	70,716				8.00	6.87, 9.32	<0.0001
Third-born	36,249				3.74	3.20, 4.36	<0.0001
Fourth-born	17,819				1.85	1.60, 2.19	<0.0001
Fifth-born	8,714				1.34	1.10, 1.63	0.003
Six+-born (ref)	8,803				1.0	–	–
**OFFSPRING SEX**							
Female (ref)	108,892				1.0	–	–
Male	118,437				1.03	1.00, 1.06	0.031

*Total N, 227,329; OR, Odds Ratio; Ref, reference group; All coefficients are calculated relative to all other variables in the table, entered into a single logistic regression model*.

**Figure 4:**
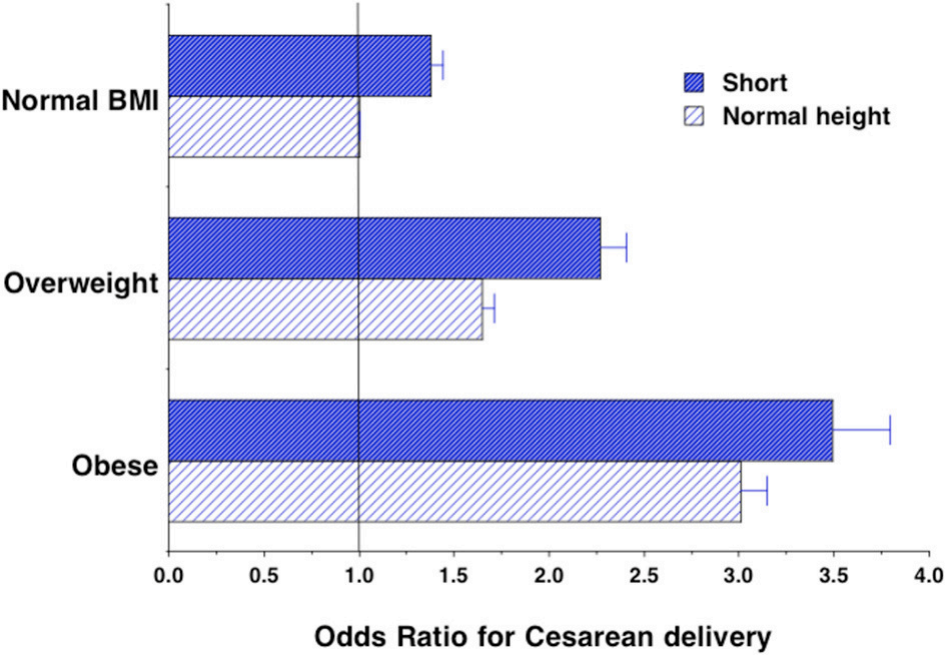
Odds of cesarean delivery associated with short stature, overweight and obesity, or their combination within mothers, in the whole sample. Results from logistical regression models, adjusted for maternal age, birth order, rural/urban status, offspring sex, offspring birth size, births within survey and wealth index. Error bars represent 95% confidence intervals.

Taking all these associations into account in the whole 2015–2016 sample, the risk of C-section was 38% greater if the mother was short with normal BMI, and 65 and 201% greater, respectively if the mother was normal height and either overweight or obese (Table 3, Figure 4). Moreover, the risks associated with overweight and obesity were further increased relative to the normal height normal BMI reference group (127 and 249%, respectively) if the mother was also short, compared to being normal stature. The OR for cesarean delivery in short-overweight mothers compared to normal-height overweight mothers was 1.37 (95%CI 1.29, 1.45), *p* < 0.0001, while that for short-obese mothers compared to normal-height obese mothers was 1.17 (95%CI 1.08, 1.27), *p* < 0.0001. The odds of C-section were 33% greater if the offspring was categorized larger than average or very large birth size, compared to average or smaller. If the analysis was restricted to the most recent pregnancy, the findings were essentially unchanged (data not shown).

The majority of the mothers (173,518; 98.1%) provided a response to the question on diabetes. Of those who did, only a small minority (*n* = 1079, 0.6%) stated that they had the condition. Those with diabetes were older than those without (Δ = 2.0 years, 95%CI 1.7, 2.3), had greater BMI (Δ = 1.8 kg/m^2^, 95%CI 1.6, 2.0) and stature (Δ = 0.5 cm, 95%CI 0.2, 0.9), and had higher average wealth index (Δ = 0.33 scores, 95%CI 0.25, 0.41). When added to the logistic regression model generated above, maternal diabetes was associated with an independent increased risk of C-section (OR 1.73, 95%CI 1.51, 1.99).

**Figure 5:**
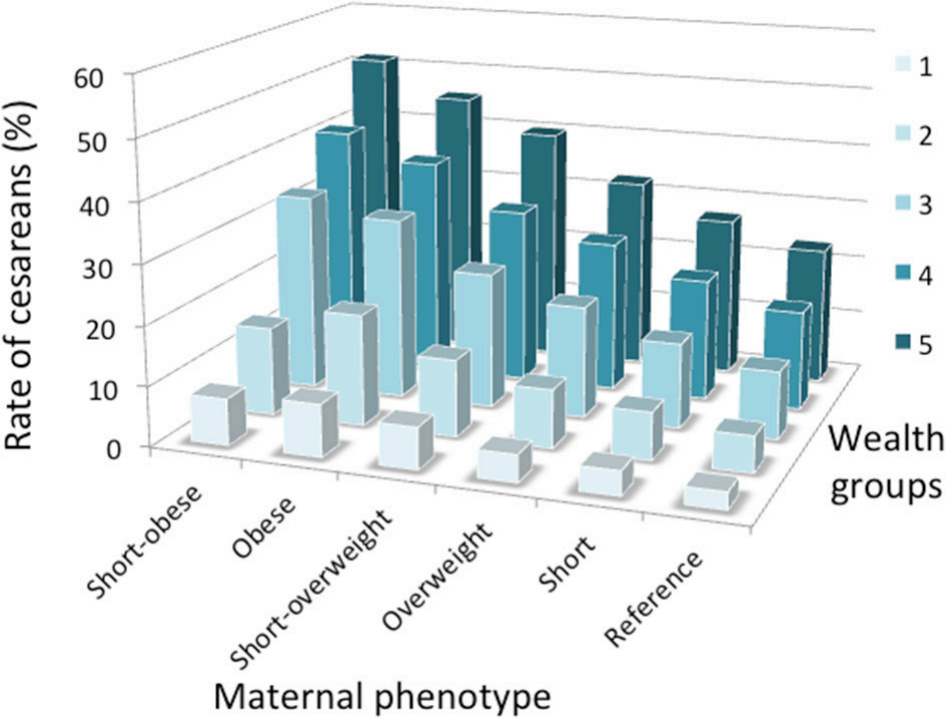
Interactive associations of wealth category and maternal somatic phenotype with the crude rate of C-section. Numbers per group are given in Table S3.

**Figure 6:**
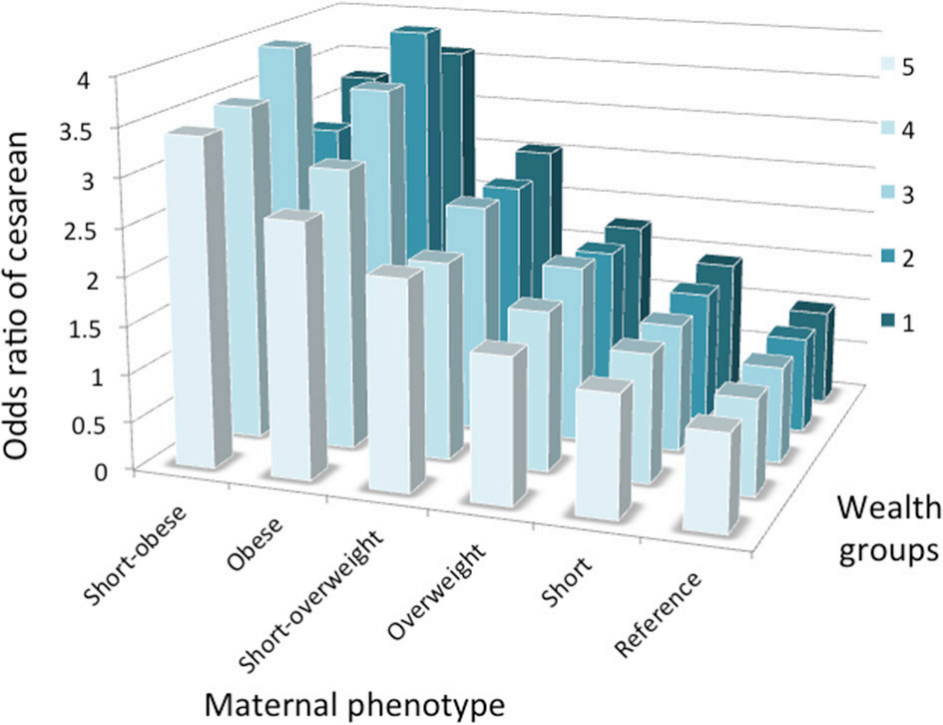
Odds of cesarean delivery associated with short stature, overweight and obesity, or their combination within mothers, stratified by wealth category (5 = wealthiest). Results from logistical regression models, adjusted for maternal age, birth order, rural/urban status and offspring sex. Numbers per group are given in Table S3.

Figure 5 illustrates the association between maternal phenotype and rate of C-section stratified by wealth group. For any category maternal phenotype, the risk of C-section rose with wealth, whereas the association between C-section and maternal phenotype seems strongest among the wealthiest group. However, as described above, confounders such as low birth order, urban location, older maternal age and male offspring also clustered more strongly among the wealthier groups. Figure 6 therefore presents odds ratios for the risk of C-section for each maternal phenotype category, stratified by wealth group and adjusting for confounders. The association of C-section risk with maternal phenotype category (short stature, overweight/obesity or their combination) was relatively similar within each wealth group, indicating that maternal phenotype was not merely a proxy for socio-economic status but was a direct predictor of C-section risk. Moreover, if the covariates are removed and unadjusted odds ratios are considered, the pattern changes minimally (Figure S1), indicating that the wealth-specific associations of maternal anthropometry with cesarean risk are relatively independent of the covariates.

### Contribution of secular trends in maternal phenotype to c-section trends

For the surveys combined, the unadjusted odds of cesarean delivery for categories of maternal phenotype were as follows: overweight 2.52 (95%CI 2.44, 2.60), *p* < 0.0001; obese 5.32 (95%CI 5.12, 5.52), *p* < 0.0001; short 0.96 (95%CI 0.93, 0.99), *p* = 0.017; short-overweight 2.48 (95%CI 2.36, 2.61), *p* < 0.0001; short-obese 5.11 (95%CI 4.76, 5.48), *p* < 0.0001. Incorporating both surveys in the regression model described in Table 3, but initially excluding maternal nutritional phenotype, women in the second survey had an increased likelihood of delivering by C-section (OR 1.49, 95%CI 1.44, 1.55; Table 4). Adding in maternal phenotype, the coefficient for the second survey decreased to 1.40 (95%CI 1.35, 1.45; Table 4). Thus, taking into account baseline C-section rate, 18% of the secular increase in C-section risk could be attributed to changes in maternal phenotype (Table S4). Again, if the analysis was restricted to the most recent pregnancy, the findings were essentially unchanged (data not shown).

**Table 4:** Multivariable logistic regression models for the odds of cesarean delivery incorporating both surveys, (a) without and (b) with adjustment for maternal somatic phenotype.

		**(a) Unadjusted for maternal phenotype**			**(b) Adjusted for maternal phenotype**		
		**Nagelkerke *r*^2^ = 0.201**			**Nagelkerke *r*^2^ = 0.224**		
**Predictor**	***N***	**OR**	**95% CI**	***p***	**OR**	**95% CI**	***p***
**SURVEY**							
2005–2006 (ref)	42,184	1.0			1.0		
2015–2016	227,329	1.49	1.44, 1.55	<0.0001	1.40	1.35, 1.45	<0.0001
**MATERNAL NUTRITIONAL STATUS**							
Normal height normal BMI (ref)	151,355				1.0	–	–
Overweight	36,842				1.65	1.62, 1.73	<0.0001
Obese	14,883				3.00	2.88, 3.13	<0.0001
Short	52,727				1.37	1.32, 1.42	<0.0001
Short overweight	10,036				2.32	2.20, 2.46	<0.0001
Short obese	3,670				3.57	3.31, 3.86	<0.0001
**MATERNAL AGE**							
15–19 years (ref)	7,853	1.0	–	–	1.0	–	–
20–24 years	82,554	1.23	1.14, 1.33	<0.0001	1.20	1.11, 1.30	<0.0001
25–29 years	102,384	1.71	1.58, 1.84	<0.0001	1.57	1.45, 1.69	<0.0001
30–34 years	50,447	2.66	2.46, 2.89	<0.0001	2.25	2.08, 2.44	<0.0001
35–39 years	19,399	3.62	3.13, 3.96	<0.0001	2.91	2.66, 3.19	<0.0001
40–44 years	5,388	4.44	3.92, 5.03	<0.0001	3.46	3.05, 3.93	<0.0001
45–49 years	1,488	3.10	2.39, 4.03	<0.0001	2.50	1.92, 3.27	<0.0001
**BIRTH NUMBER WITHIN SURVEY**							
Most recent birth within 5 years (ref)	203,546	1.0	–	–	1.0	–	–
2nd most recent birth within 5 years	59,484	0.49	0.47, 0.50	<0.0001	0.49	0.48, 0.51	<0.0001
3rd most recent birth within 5 years	6,483	0.22	0.19, 0.25	<0.0001	0.22	0.19, 0.25	<0.0001
**WEALTH INDEX**							
Level 1 (ref)	66,038	1.0	–	–	1.0	–	–
Level 2	61,064	1.89	1.79, 1.99	<0.0001	1.82	1.72, 1.92	<0.0001
Level 3	54,414	3.42	3.25, 3.59	<0.0001	3.14	2.98, 3.30	<0.0001
Level 4	47,858	4.71	4.48, 4.95	<0.0001	4.15	3.94, 4.36	<0.0001
Level 5 (wealthiest)	40,139	6.18	5.87, 6.51	<0.0001	5.12	4.85, 5.40	<0.0001
**RESIDENCE**							
Rural (ref)	199,457	1.0	–	–	1.0	–	–
Urban	70,056	1.26	1.23, 1.30	<0.0001	1.20	1.17, 1.23	<0.0001
**BIRTH ORDER**							
First-born	98,478	16.9	14.7, 19.5	<0.0001	16.38	14.2, 18.84	<0.0001
Second-born	82,692	9.37	8.2, 10.8	<0.0001	8.92	7.76, 10.25	<0.0001
Third-born	43,103	4.33	3.76, 4.98	<0.0001	4.10	3.56, 4.72	<0.0001
Fourth-born	21,920	2.07	1.78, 2.42	<0.0001	2.00	1.71, 2.33	<0.0001
Fifth-born	11,193	1.46	1.22, 1.75	<0.0001	1.43	1.20, 1.71	<0.0001
Sixth+-born (ref)	12,127	1.0	–	–	1.0	–	–
**OFFSPRING SEX**							
Female (ref)	129,076	1.0	–	–	1.0	–	–
Male	140,437	1.03	1.00, 1.05	0.016	1.04	1.01, 1.06	0.003

*Total N, 275,271; OR, Odds Ratio; Ref, reference group. All coefficients are calculated relative to all other variables in the table, entered into a single logistic regression model*.

## Discussion

This study has described secular trends in C-section rate in India, and tested whether this might be related to maternal short stature and overweight/obesity, taking into account confounders such as birth order category, wealth status, rural/urban location, offspring sex and maternal age. Previous analyses have linked both short stature and maternal overweight with the risk of C-section, but have not considered them in combination.

Our analysis produced a number of key findings. First, controlling for changes in the profile of the women sampled, we have quantified a 49% secular increase in the odds of C-section in India over a 10 years period. This was associated with a substantial secular increase in BMI over the last 10 years among women of reproductive age but negligible increase in height, indicating that weight increased out of proportion to height. We further documented a secular increase in offspring birth size, though our analysis was crude, being limited to categorical assessments of size.

Second, we documented inter-relationships between several risk factors for C-section, such as between wealth and maternal somatic phenotype or birth order. This allowed us to describe interactive associations, for example both birth order and wealth were associated with the C-section rate, with the highest rates occurring among wealthy first-time mothers. The secular increase in C-sections was most evident in mothers of birth order 1 to 3, and among the middle wealth groups.

Third, consistent with our hypothesis, we found that the risk of C-section was elevated in short women compared to women of normal stature. Likewise, we found that the risk of C-section was raised in overweight women, and even more so among obese women. Finally, we showed that the elevated risk of C-section in overweight or obese women was further increased, if they were also short. Thus, the two maternal risk factors generated a greater risk when they occurred jointly compared to when they occurred separately. Analyzing both surveys combined, 18% of the secular increase in C-section rate was attributable to the secular trends in maternal phenotype. Maternal diabetes also was independently associated with risk of C-section, though the magnitude of this effect should be considered with caution due to the small number of diabetic women in the sample. While wealthier women were both more likely to be obese and potentially more likely to have access to the medical facilities required for C-section, we found that the association between maternal somatic phenotype and C-section risk was very similar across the 5 wealth categories.

Overall, our findings indicate that the emerging “dual burden” of malnutrition, where child malnutrition and adult short stature persist even as overweight emerges, is likely to have a major detrimental effect on childbirth, impacting morbidity and mortality risk of both mothers and offspring. The nature of economic development and associated nutrition transition is closely associated with the emerging obesity epidemic (34), but child under-nutrition remains very prevalent (35) and any secular trend in height is very modest in India compared to other global regions (36). So far, research and policy attention to the health implications of the dual burden has focused on non-communicable diseases, such as diabetes and cardiovascular disease (34).

South Asian countries are well established to have high rates of low birth weight, child stunting and short adult stature compared to other countries (35–37), and also low age at first birth due to a high prevalence of early marriage (38). Persistent child malnutrition is a key contributing factor to short adult stature, demonstrated by inverse dose-response associations between the magnitude of child stunting and adult female height in India (39), though other factors are also relevant. At an absolute level, mean BMI is relatively low in South Asian countries compared to other populations, but this is in large part due to low levels of lean mass (40). Using ethnic-specific BMI-cut-offs for Asian populations proposed by WHO (26), overweight and obesity are increasingly prevalent in India (41), and hence the dual burden of malnutrition is already severe. Although secular increases in height may propagate to secular increases in pelvic dimensions (21), the negligible magnitude of such height trends in the Indian population (36) indicates that short stature remains a significant constraint on childbirth. However, efforts to delay the age at marriage, and hence the age of first childbirth, might at least delay childbirth until pelvic growth is approaching completion.

Given ethnic and geographical differences in height and BMI, the magnitude of associations between maternal short stature and overweight with C-section that we report here may not generalize to all other countries. Nevertheless, both short stature and overweight are common traits in many populations, and our analyses broadly suggest that the global trend toward a dual burden of malnutrition, in which the obesity epidemic is emerging even as the inter-generational consequences of chronic under-nutrition persist, will generate a major impact on maternal and child health.

From an evolutionary perspective, others have suggested that high rates of obstetric mortality favor larger female size, detected as a lower level of sexual dimorphism in adult height (42). However, India has experienced an unusually large secular decline in height in the last 10,000 years (43), and this is likely to contribute to low average birth weights in the contemporary population (44). Analyzing the offspring of inter-ethnic unions within the UK, we found that both Indian paternity and maternity are associated with lower offspring birth weight, compared to European parentage, indicating a degree of genetic adaptation of fetal growth within Indians to small maternal body size (45).

From a public health perspective, our study highlights elevated risk of C-section in mothers who are short, overweight, older, and who are giving birth to their first offspring. The last three of these factors are becoming more typical of mothers through secular trends in nutrition and fertility patterns. Other research, including a study in India, reported that pelvic dimensions are still increasing even after height has reached its maximum (32). On that basis, the low risks we found associated with younger maternal age may seem counter-intuitive. However, this effect emerges in concert with the very high risk associated with first-time deliveries, which is closely associated with young age.

The strengths of our analysis include the use of two large surveys measured with a common protocol, and the availability of substantial data on potential confounders, allowing us to assess secular trends in our key exposures and outcome despite some differences in sampling distribution across the two surveys. The large sample size also enabled us to look within categories of wealth, to clarify the direct association of maternal phenotype with C-section risk.

Limitations include the cross-sectional nature of the data, the lack of more detailed information about birth complications, and the crude categorization of size at birth. We do not have direct data on reliability of the surveys, however the anthropometric data were obtained by objective measurements, and any measurement error should be negligible relative to the range of height and BMI in the population, resulting in a high likelihood of individuals being allocated to the appropriate height and BMI categories. The cesarean data were obtained using the protocol advocated to maximize reliability in DHS surveys (31). Recent validation studies of cesarean delivery in China, Ghana and the Dominican Republic reported sensitivity/specificity values of 96%/83%, 79%/82%, and 50%/80% respectively (46, 47).

The maternal anthropometric data were collected up to a maximum of 5 years after the time of the first birth included in the analyses, hence the mother could have changed in weight (and potentially also height, if adolescent) since the first birth. However, we consider this issue has not introduced artifacts into our analysis, as the regression models produced essentially unchanged findings if the sample was restricted to only the most recent birth of each mother, which would reduce any such temporal lag. It is also possible that cesarean delivery might itself affect the subsequent pattern of maternal weight gain, though evidence to support this hypothesis is lacking (48). We therefore consider it unlikely that our finding that maternal obesity increases cesarean risk incorporates any substantial effect of reverse causation. Our analysis might also be affected by survival bias, as mothers experiencing the greatest complications of childbirth may have died at that time, or may not have participated in the DHS survey due to health issues.

Our findings suggest that maternal phenotype should be taken into account when considering whether the incidence of C-section is inadequate or excessive. While the relative contributions of short stature and overweight/obesity differed between the poor rural women compared to the wealthy urban women in this sample, both populations contain high numbers of women at risk. The poorest rural woman had high rates of short stature but relatively low rates of obesity. That only 3.5% of these rural women delivered by C-section suggests that this part of the population continues to lack adequate access to surgical care, increasing the risk of death due to obstructed labor. In contrast, the wealthiest women had low rates of short stature and high rates of overweight/obesity. A third of these mothers delivered by C-section, a level much higher than that of 10–15% recommended by WHO (2).

Nevertheless, WHO recommendations take no account of variability in the two maternal risk factors identified here. Populations with high prevalences of short overweight women may need C-section rates higher than 10–15% in order to minimize maternal and neonatal mortality. Our study cannot address this, and further work is required to investigate this issue in more detail. We do not contradict concern that C-section rates may be excessive in many populations (49), rather we offer a complementary message that the rate of C-section may potentially vary across populations, in association with variability in maternal height and weight.

## Ethics statement

We conducted secondary analysis of fully anonymized DHS data which is freely available for academic research. The DHS website provides relevant information, which is included in our manuscript on page 2.

## Author contributions

JW conceived the study, ran the main statistical analyses and wrote the first draft of the manuscript. RW contributed to statistical analysis and commented on the draft manuscript. MP extracted and coded the data and commented on the statistical analyses and draft manuscript.

### Conflict of interest statement

The authors declare that the research was conducted in the absence of any commercial or financial relationships that could be construed as a potential conflict of interest.
