# Supplementary material for: The Dual Burden of Malnutrition Increases the Risk of Cesarean Delivery: Evidence From India
Source: Front Public Health. 2018 Oct 17;6:292. doi: 10.3389/fpubh.2018.00292 (PMC6199394; doi:10.3389/fpubh.2018.00292)

Figure S1. Odds of cesarean delivery associated with short stature, overweight and obesity, or their combination within mothers, stratified by wealth category (5 = wealthiest).

Results from logistical regression models, unadjusted for any covariates.
Numbers per group are given in Table S3

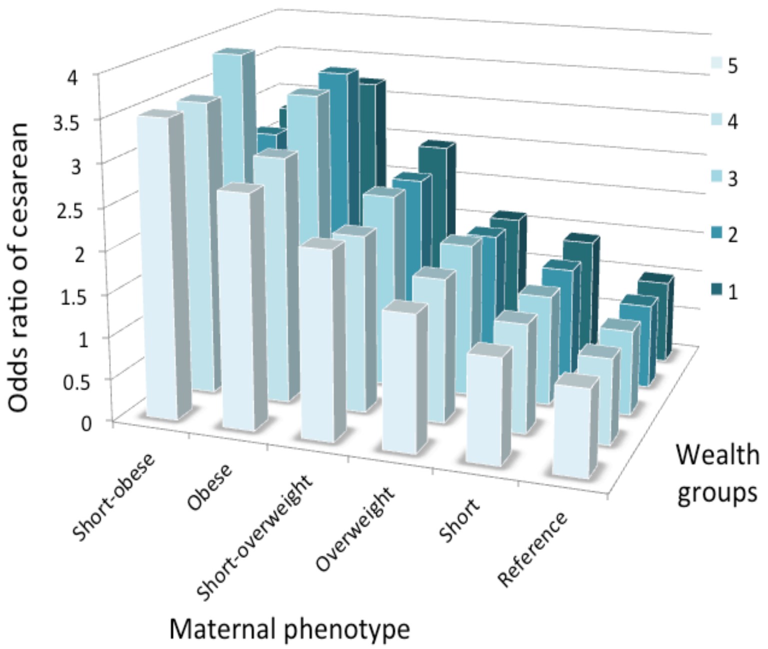

Supplement: Supplementary file 1 [file Image_1.pdf]
