# Supplementary material for: The Dual Burden of Malnutrition Increases the Risk of Cesarean Delivery: Evidence From India
Source: Front Public Health. 2018 Oct 17;6:292. doi: 10.3389/fpubh.2018.00292 (PMC6199394; doi:10.3389/fpubh.2018.00292)
Supplement: Supplementary file 2 [file Table_1.pdf]

Table S1. Distribution of maternal phenotype and key confounding factors by wealth status in the 2015-2016 survey

**Wealth category**	**1**	**2**	**3**	**4**	**5**	P for Chi-square
**Maternal phenotype**						
Short, normal BMI (%)	30.6	22.9	16.2	10.5	5.5	<0.0001
Normal height overweight (%)	6.0	10.7	16.0	21.3	27.2	
Normal height obese (%)	1.0	2.6	5.8	10.1	16.6	
Short overweight (%)	3.3	4.2	4.6	4.4	3.4	
Short obese (%)	0.5	1.0	1.8	2.5	2.7	
**Location**						
Urban	4.5	10.4	22.5	43.1	62.6	<0.0001
**Birth order** *						
First-born	16.4	20.6	21.0	20.8	21.2	<0.0001
**Maternal age** *						
15-19 y	3.7	4.1	3.2	2.1	1.0	<0.0001
**Child sex**						
Male	53.8	54.1	54.6	53.8	55.5	<0.0001

Selected groups only displayed
N = 175,790 mothers, 232,411 deliveries
