# Supplementary material for: The Dual Burden of Malnutrition Increases the Risk of Cesarean Delivery: Evidence From India
Source: Front Public Health. 2018 Oct 17;6:292. doi: 10.3389/fpubh.2018.00292 (PMC6199394; doi:10.3389/fpubh.2018.00292)
Supplement: Supplementary file 3 [file Table_2.pdf]

Table S2. Multivariate logistic regression model for the odds of cesarean delivery in the 2006 survey.

	N=42,184, Nagelkerke r^2 = 0.258		
**Predictor**	**Odds ratio**	**95% CI**	**p**
**Maternal nutritional status**			
Normal height normal BMI	-	-	-
Overweight	1.69	1.53, 1.86	<0.0001
Obese	2.63	2.33, 2.97	<0.0001
Short	1.41	1.28, 1.56	<0.0001
Short overweight	2.76	2.36, 3.23	<0.0001
Short obese	4.26	3.36, 5.40	<0.0001
**Offspring birth size**			
Average size or smaller	-	-	-
Larger than average or very large	1.21	1.12, 1.30	<0.0001
**Maternal age**			
15-19 years	-	-	-
20-24 years	1.32	1.10, 1.59	0.003
25-29 years	1.97	1.63, 2.37	<0.0001
30-34 years	2.92	2.40, 3.57	<0.0001
35-39 years	4.18	3.32, 5.26	<0.0001
40-44 years	4.59	3.21, 6.55	<0.0001
45-49 years	1.16	0.38, 3.58	0.7
**Birth number within survey**			
Most recent birth within 5 years	-	-	-
2nd most recent birth within 5 years	0.53	0.48, 0.58	<0.0001
3rd most recent birth within 5 years	0.26	0.18, 0.37	<0.0001
**Wealth index**			
Level 1	-	-	-
Level 2	2.04	1.61, 2.58	<0.0001
Level 3	3.38	2.71, 4.20	<0.0001
Level 4	5.21	4.21, 6.46	<0.0001
Level 5	7.38	5.93, 9.17	<0.0001
**Residence**			
Rural	-	-	-
Urban	1.28	1.19, 1.39	<0.0001
**Birth order**			
First-born	22.9	16.1, 32.6	<0.0001
Second-born	11.2	7.9, 15.8	<0.0001
Third-born	4.98	3.48, 7.12	<0.0001
Fourth-born	2.29	1.55, 3.38	<0.0001
Fifth-born	1.61	1.02, 2.54	0.041
Six+-born	-	-	-
**Offspring sex**			
Female	-	-	-
Male	1.05	0.98, 1.12	0.18

All coefficients are calculated relative to all other variables in the table, entered into a single logistic regression model
