# Supplementary material for: The Dual Burden of Malnutrition Increases the Risk of Cesarean Delivery: Evidence From India
Source: Front Public Health. 2018 Oct 17;6:292. doi: 10.3389/fpubh.2018.00292 (PMC6199394; doi:10.3389/fpubh.2018.00292)
Supplement: Supplementary file 4 [file Table_3.pdf]

Table S3. Numbers included per group in Figure 3 and Figures 5 and 6

(a) Numbers per group in Figure 3

	Birth order					
Wealth index	1	2	3	4	5	6
1	16002	15970	11994	7638	4360	4964
2	18750	16461	9760	5037	2526	2561
3	18430	15277	7195	3093	1323	1164
4	17273	13406	5120	1853	969	498
5	15838	11021	3302	882	236	132

(b) Numbers per group in Figures 5, 6 and S1

	Wealth index				
Maternal phenotype	1	2	3	4	5
Normal weight and height	35,654	32,384	26,125	20,159	14,104
Short	18,948	12,775	7,747	4,263	1,815
Overweight	3,483	5,761	7,239	8,056	8,471
Short-overweight	1,960	2,228	2,078	1,728	1,079
Obese	558	1,327	2,526	3,741	5,117
Short-obese	321	528	766	899	824
