# Supplementary material for: The Dual Burden of Malnutrition Increases the Risk of Cesarean Delivery: Evidence From India
Source: Front Public Health. 2018 Oct 17;6:292. doi: 10.3389/fpubh.2018.00292 (PMC6199394; doi:10.3389/fpubh.2018.00292)
Supplement: Supplementary file 5 [file Table_4.pdf]

Table S4. Calculation of the change in C-section rates across surveys adjusting for confounders, and the contribution of trends in maternal phenotype to this change

The odds ratio of C-section in the second ratio can be calculated using the equation:

$$OR = [P2/Q2] / [P1/Q1]$$

where P is the fraction of the population delivering by C-section, Q is the remaining fraction of the population, and the values 1 and 2 refer to the two surveys in appropriate order.

Rearranging this equation in combination with algebra, P2 can be calculated from the OR using the equation:

$$P2 = [P1 * OR] / [1 + (P1 * (OR - 1))]$$

Without adjusting for maternal phenotype, the OR for C-section for the second survey after adjustment for confounders is 1.49 (see table 4, main article). On this basis, the rate of C-sections increased by 4.4%, from 10.4% to 14.8%.

The OR for C-section for the second survey after adjustment for both confounders and maternal phenotype is 1.40 (see Table 4, main article). On this basis, the rate of C-sections increased across the surveys by 3.6%, from 10.4% to 14.0%.

Therefore 18% (ie 0.8/4.4) of the secular increase in the rate of C-sections is explained by secular changes in maternal phenotype, of which the primary component is increases in maternal overweight/obesity.

These equations were kindly provided by Professor Tim Cole, UCL Great Ormond Street Institute of Child Health
